# Evaluating auto‐contouring accuracy in reduced CT dose images for radiopharmaceutical therapies: Denoising and evaluation of ^177^Lu DOTATATE therapy dataset

**DOI:** 10.1002/acm2.70066

**Published:** 2025-03-02

**Authors:** Hung‐Te Yang, Kuan‐Yin Ko, Ching‐Ching Yang

**Affiliations:** ^1^ Department of Radiation Oncology Kaohsiung Municipal Siaogang Hospital Kaohsiung Taiwan; ^2^ Department of Nuclear Medicine National Taiwan University Cancer Center Taipei Taiwan; ^3^ Department of Medical Imaging and Radiological Sciences Kaohsiung Medical University Kaohsiung Taiwan; ^4^ Department of Medical Research Kaohsiung Medical University Hospital Kaohsiung Taiwan

**Keywords:** auto contouring, CNN‐based denoising, Lu‐177 DOTATATE therapy

## Abstract

**Purpose:**

Reducing radiation dose attributed to computed tomography (CT) may compromise the accuracy of organ segmentation, an important step in ^177^Lu DOTATATE therapy that affects both activity and mass estimates. This study aimed to facilitate CT dose reduction using deep learning methods for patients undergoing serial single photon emission computed tomography (SPECT)/CT imaging during ^177^Lu DOTATATE therapy.

**Methods:**

The ^177^Lu DOTATATE patient dataset hosted in Deep Blue Data was used in this study. The noise insertion method incorporating the effect of bowtie filter, automatic exposure control, and electronic noise was applied to simulate images at four reduced dose levels. Organ segmentation was carried out using the TotalSegmentator model, while image denoising was performed with the DenseNet model. The impact of segmentation performance on the dosimetry accuracy of ^177^Lu DOTATATE therapy was quantified by calculating the percent difference between a dose rate map segmented with a reference mask and the same dose rate map segmented with a test mask (PD_dose_) for spleen, right kidney, left kidney, and liver.

**Results:**

Before denoising, the mean ± standard deviation of PD_dose_ for all critical organs were 2.31 ± 2.94%, 4.86 ± 9.42%, 8.39 ± 14.76%, 12.95 ± 19.99% in CT images at dose levels down to 20%, 10%, 5%, 2.5% of the normal dose, respectively. After denoising, the corresponding results were 1.69 ± 2.25%, 2.84 ± 4.46%, 3.72 ± 4.22%, 7.98 ± 15.05% in CT images at dose levels down to 20%, 10%, 5%, 2.5% of the normal dose, respectively.

**Conclusion:**

As dose reduction increased, CT image segmentation gradually deteriorated, which in turn deteriorated the dosimetry accuracy of ^177^Lu DOTATATE therapy. Improving CT image quality through denoising could enhance ^177^Lu DOTATATE dosimetry, making it a valuable tool to support CT dose reduction for patients undergoing serial SPECT/CT imaging during treatment.

## 1 INTRODUCTION

Somatostatin receptor based ^177^Lu DOTATATE therapy is a promising approach to treat neuroendocrine tumors. Compared to conventional radiation therapy, peptide receptor radionuclide therapy encompasses a unique targeting mechanism to efficiently deliver ionizing radiation to disseminated cancer cells and small metastases.^1^, ^2^, ^3^ However, physiological uptake of ^177^Lu DOTATATE can also be seen in liver, spleen, kidneys, and bone marrow.^4^, ^5^, ^6^ While bone marrow dosimetry could be calculated using the venous blood samples, image‐based dosimetry is a common method for calculating the absorbed and effective doses for liver, spleen, and kidneys, in which serial imaging has to be performed after ^177^Lu DOTATATE administration to determine radioactivity in the critical organs over time.^7^, ^8^, ^9^ single photon emission computed tomography (SPECT) imaging‐based dosimetry outperformed whole‐body planar imaging‐based dosimetry because organs at risk can be quantified with minimal signal overlap from surrounding structures.^10^, ^11^, ^12^ Organ segmentation plays an important role in ^177^Lu DOTATATE dosimetry, and contouring on computed tomography (CT) images is a practical approach used in clinical practice. The current standard in ^177^Lu DOTATATE therapy is to administer four 7.4 GBq cycles. If serial SPECT/CT are acquired at 4, 24, and 96 h postinjection per cycle, patients receive 12 CT scans throughout the whole treatment. According to Sharma et al., the CT effective dose was 3.9 ± 0.6 mSv, 4.6 ± 2.5 mSv, 4.8 ± 2.7 mSv for SPECT/CT of the chest, abdomen, and pelvis, respectively.^13^ Cao et al. evaluated whether adult CT scan exposure can increase the risks of cancer during the follow‐up observation and found that cancer risks increased rapidly during radiation dose above 55 mSv.^14^ Hence, the consequent CT‐related radiation exposure should be considered. Kan et al. investigated the radiation dose from cone beam CT for image‐guided radiation therapy. They suggested that patient position verification by standard mode cone beam CT could increase the secondary cancer risk by up to 2% to 4%, so lower mAs settings for daily cone beam CT should be considered.^15^ Radiation dose is directly proportional to the mAs, so tube current reduction is the most accessible method of reducing CT radiation dose.^16^ However, this process would increase image noise and degrade target detectability since the quantum noise is inversely proportional to the square root of mAs.^17^ The resultant effects may deteriorate the accuracy of organ segmentation in CT images, thus impacting ^177^Lu DOTATATE dosimetry for critical organs. Deep learning gets a lot of attention recently to solve various problems in the medical imaging field.^18^, ^19^, ^20^, ^21^, ^22^ The outcomes of a deep learning segmentation model proposed by Wasserthal et al. showed a slight difference between normal‐dose CT (NDCT) and low‐dose CT (LDCT).^23^ Deep learning also offers solutions to map LDCT back to the corresponding NDCT representations.^24^, ^25^ This study aimed to facilitate CT dose reduction using deep learning methods for patients undergoing serial SPECT/CT imaging during ^177^Lu DOTATATE therapy.

## 2 METHODS

### 2.1 Patient dataset

**FIGURE 1:**
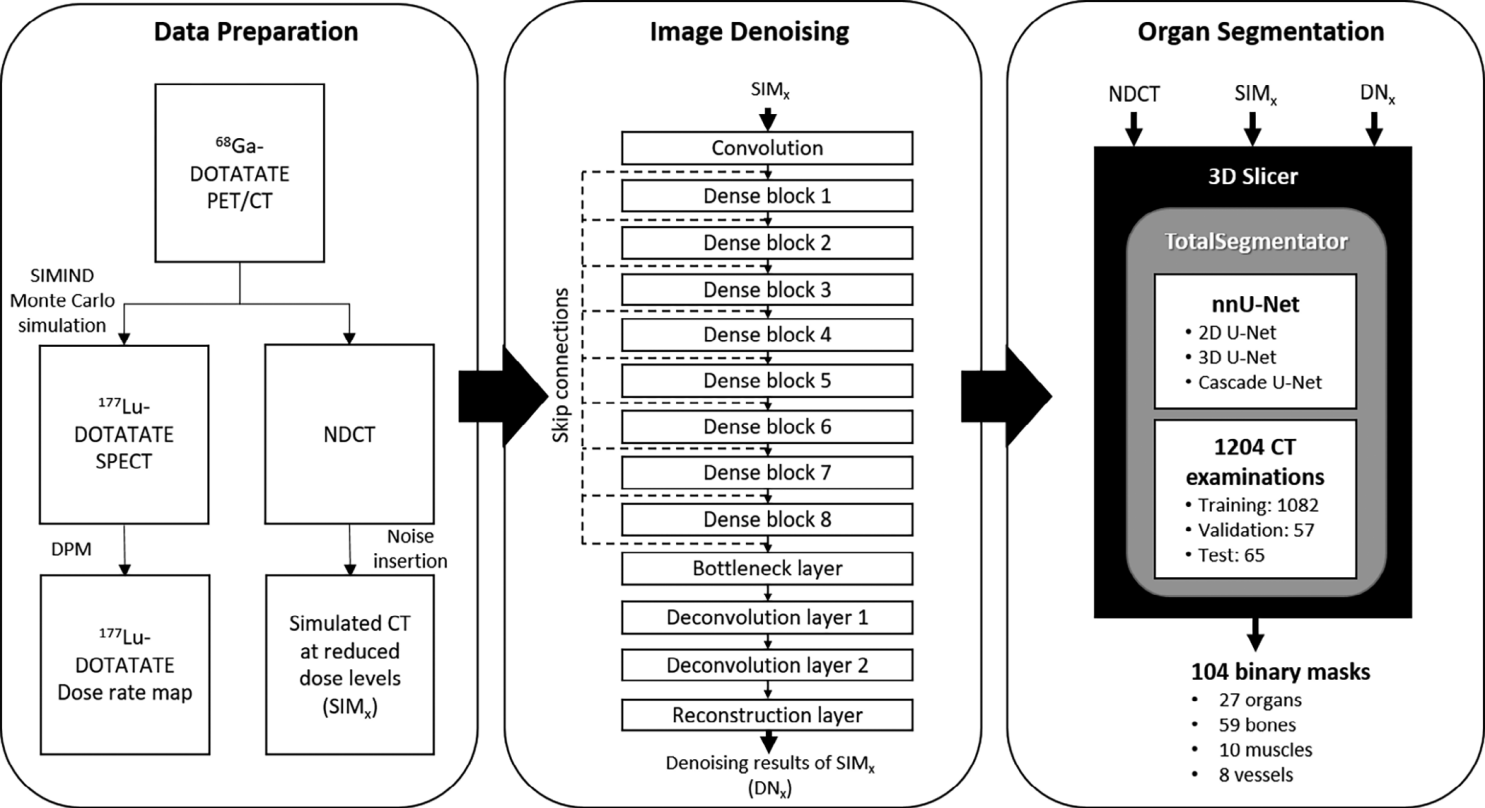
Illustration of our flowchart for data preparation, image denoising, and organ segmentation (x = 20%, 10%, 5%, 2.5%).

The ^177^Lu DOTATATE patient dataset hosted in Deep Blue Data, a repository for sharing and preserving research data developed at the University of Michigan, was used in this study.^26^ The dataset consists of 16 patients who underwent diagnostic ^68^Ga DOTATATE PET/CT imaging using a Biograph mCT TOF PET/CT scanner (Siemens Healthineers, Erlangen, Germany) to determine eligibility for therapy. The ^177^Lu SPECT projections were generated via SIMIND Monte Carlo simulation with activity maps derived from ^68^Ga PET images and density maps derived from CT images. The SIMIND model parameters were based on ^177^Lu SPECT imaging acquired using Symbia Intevo SPECT/CT system (Siemens Healthineers, Erlangen, Germany) with medium energy collimators, a 5/8‐inch crystal, a 20% photopeak window at 208 keV, and two adjacent 10% scatter windows. The ^177^Lu SPECT images were reconstructed using 3D OS‐EM (16 iterations and 4 subsets) with CT‐based attenuation correction, triple energy window scatter correction, and collimator‐detector response modeling. The matrix size and the voxel size of the SPECT images were 128 × 128 × 81 and 4.8 × 4.8 × 4.8 mm^3^, respectively. The dose rate maps were obtained by running one billion histories of dose planning method (DPM), which was optimized specifically for voxel‐level electron/photon dose computations with full radiation transport, with SPECT images.^27^ The CT images obtained from Siemens Biograph mCT were treated as standard dose examinations, referred to as NDCT. The noise insertion method incorporating the effect of bowtie filter, automatic exposure control, and electronic noise proposed by Yu et al. was applied to simulate CT images at dose levels down to 20%, 10%, 5%, and 2.5% of the normal dose.^28^ The CT images at various dose levels were then passed to 3D Slicer image computing platform for organ segmentation with an extension called TotalSegmentator, which was created at University Hospital Basel using the nnU‐Net framework developed at DKFZ (Deutsches Krebsforschungszentrum).^23^ Figure 1 illustrates the flowchart for data preparation, image denoising, and organ segmentation. Four simulated CT images at reduced dose levels (SIM_x_), the denoising results of CT images at reduced dose levels (DN_x_), and their masks were generated for the same ^177^Lu DOTATATE dose rate map. The impact of SPECT data acquisition and reconstruction on the quantification accuracy of ^177^Lu DOTATATE therapy was not investigated in this work.

### 2.2 Image denoising

The image denoising model employed in this study was DenseNet, which comprised one convolution layer to learn low level features, eight dense blocks to extract high level features, one bottleneck layer to maintain compactness and reduce computation cost, two deconvolution layers to learn upscaling filters, and one reconstruction layer to generate the output images (Figure 1).^29^ There were eight convolution layers in each dense block, and all levels of features were combined via skip connections as input for reconstructing the output images. CT images at four reduced dose levels were used as input to the DenseNet model, with the NDCT images serving as the label data. The root mean square error (RMSE) was the loss function adopted to minimize the difference between input and label images. By utilizing RMSE as the loss function, the preference is given to achieving a high peak signal‐to‐noise ratio (PSNR). The filter weights for each layer were initialized using the MSRA (Microsoft Research Asia) filler technique, while all biases were initialized to zero.^30^ The DenseNet model was trained using the Adam (adaptive moment estimation) optimizer with a mini‐batch size of 32, a learning rate of 0.0001, a momentum of 0.9, and a weight decay of 0.0001.^31^ The dataset was divided into a training set with eight patients and a test set with eight patients. The training data consisted of approximately 452 928 sub‐images, each measuring 25 × 25 pixels. These sub‐images were randomly cropped from the original images, serving as both input and label images. The DenseNet models were built by using Caffe (Convolutional Architecture for Fast Feature Embedding) deep learning platform on an Ubuntu server.^32^

### 2.3 Data analysis

RMSE and PSNR were calculated to quantify the difference between a reference image and a test image. The mathematical expression of RMSE was given by the following equation:

(1) $$\mathrm{RMSE}\;=\sqrt{\frac{\sum_{M,N}{\left|\mathrm{IM}G_{\mathrm{NDCT}}-\mathrm{IMG}\right|}^{2}}{M\cdot N}}\;$$

where $\mathrm{IM}G_{\mathrm{NDCT}}$ was NDCT with matrix size of M·N, and IMG was the corresponding CT images at reduced dose levels before and after denoising. The mathematical expression of PSNR was given by the following equation:

(2) $$\mathrm{PSNR}\;=20\cdot \log\left(\frac{HU_{\max}}{\mathrm{RMSE}}\right)$$

where HU_max_ was the maximum Hounsfield unit (HU) value of the image. The mean and standard deviation (SD) in HU were calculated for spleen, right kidney, left kidney, and liver to measure the variation of HU values within segmented regions in CT images at various dose levels before and after denoising. The accuracy of organ segmentation in CT images was assessed by calculating the percent difference between a reference mask and a test mask (PD_mask_) for spleen, right kidney, left kidney, and liver.^33^ The mathematical expression of PD_mask_ was defined as follows:

(3) $$\;PD_{\mathrm{mask}}=\frac{\sum_{\mathrm{all}\;\mathrm{pixels}}\left|BW_{\mathrm{NDCT}}-\mathrm{BW}\right|}{\sum_{\mathrm{all}\;\mathrm{pixels}}BW_{\mathrm{NDCT}}}\cdot 100\%$$

where BW_NDCT_ was the binary mask of NDCT, and BW was the binary mask of CT images at reduced dose levels before and after denoising. The impact of segmentation performance on the dosimetry accuracy of ^177^Lu DOTATATE therapy was quantified by calculating the percent difference between a dose rate map segmented with a reference mask and the same dose rate map segmented with a test mask (PD_dose_) for spleen, right kidney, left kidney, and liver.^34^ The mathematical expression of PD_dose_ was defined as follows:

(4) $$\begin{array}{ccc} & & \;PD_{\mathrm{dose}}\\ & & =\frac{\sum_{\mathrm{all}\;\mathrm{pixels}}\left|BW_{\mathrm{NDCT}}\cdot \mathrm{doseMC}-\mathrm{BW}\cdot \mathrm{doseMC}\right|}{\sum_{\mathrm{all}\;\mathrm{pixels}}BW_{\mathrm{NDCT}}\cdot \mathrm{doseMC}}\cdot 100\% \end{array}$$

where doseMC was the dose rate map obtained by running DPM with SPECT image.

## 3 RESULTS

**FIGURE 2:**
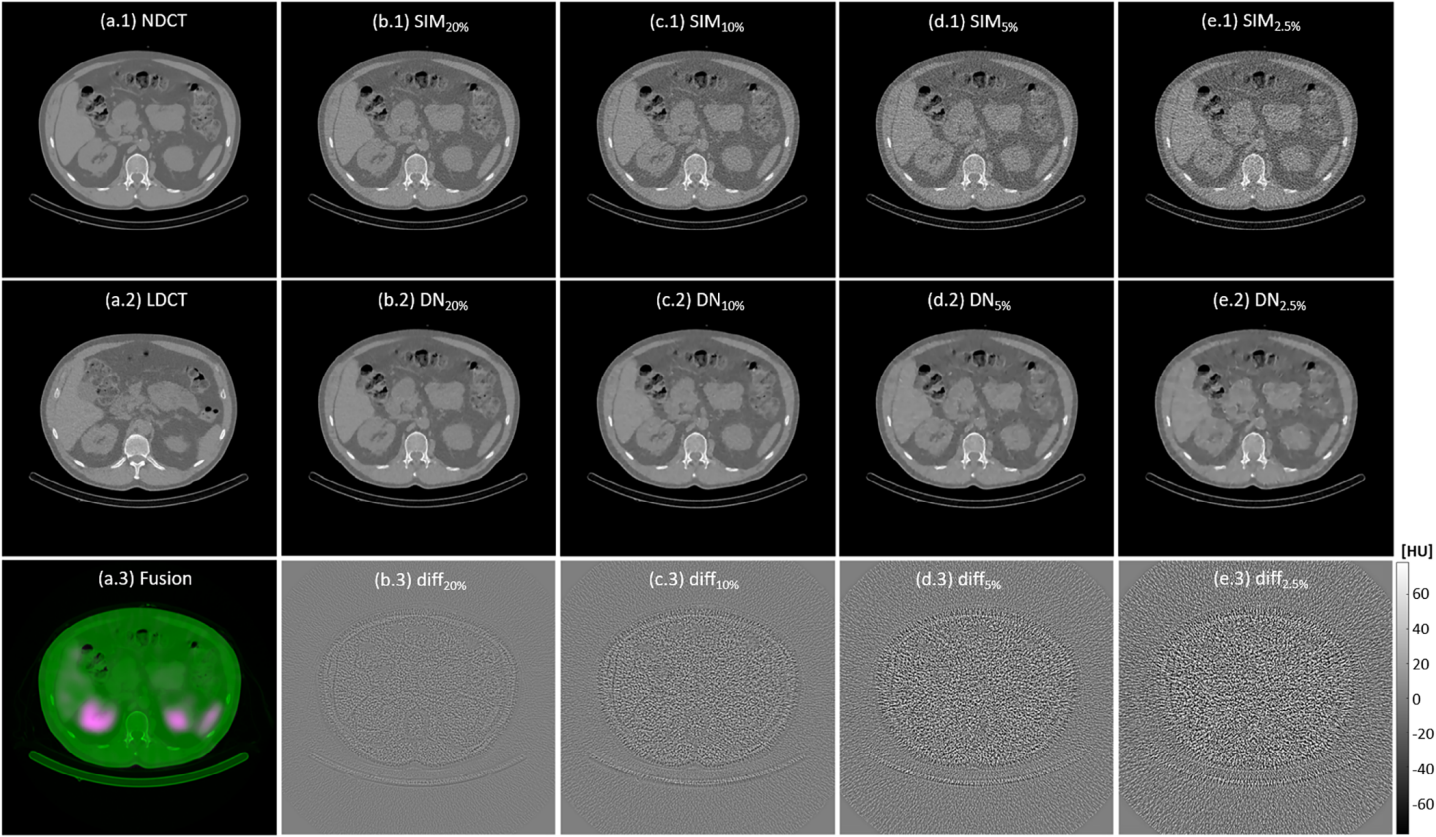
(a.1‐a.3) NDCT, LDCT, a fusion of SPECT (green) and NDCT (magenta); (b.1‐b.3) SIM_20%_, DN_20%_ and their difference (diff_20%_); (c.1‐c.3) SIM_10%_, DN_10%_ and their difference (diff_10%_); (d.1‐d.3) SIM_5%_, DN_5%_ and their difference (diff_5%_); (e.1‐e.3) SIM_2.5%_, DN_2.5%_ and their difference (diff_2.5%_).

**FIGURE 3:**
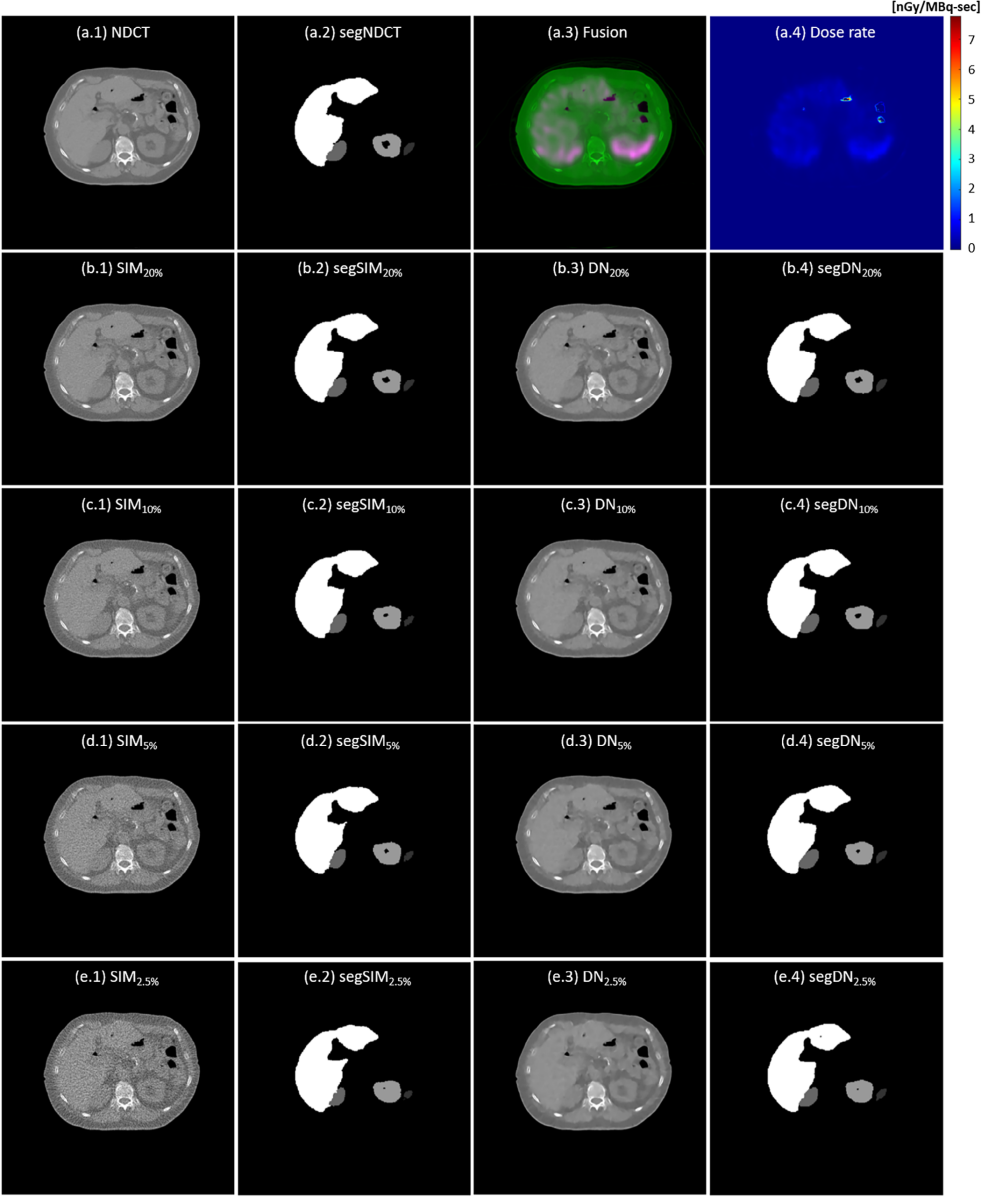
(a.1‐a.4) NDCT, segmentation mask of NDCT (segNDCT), fusion of SPECT (green) and NDCT (magenta), dose rate map; (b.1‐b.4) SIM_20%_, segmentation mask of SIM_20%_ (segSIM_20%_), DN_20%_, segmentation mask of DN_20%_ (segDN_20%_); (c.1‐c.4) SIM_10%_, segmentation mask of SIM_10%_ (segSIM_10%_), DN_10%_, segmentation mask of DN_10%_ (segDN_10%_); (d.1‐d.4) SIM_5%_, segmentation mask of SIM_5%_ (segSIM_5%_), DN_5%_, segmentation mask of DN_5%_ (segDN_5%_); (e.1‐e.4) SIM_2.5%_, segmentation mask of SIM_2.5%_ (segSIM_2.5%_), DN_2.5%_, segmentation mask of DN_2.5%_ (segDN_2.5%_).

**FIGURE 4:**
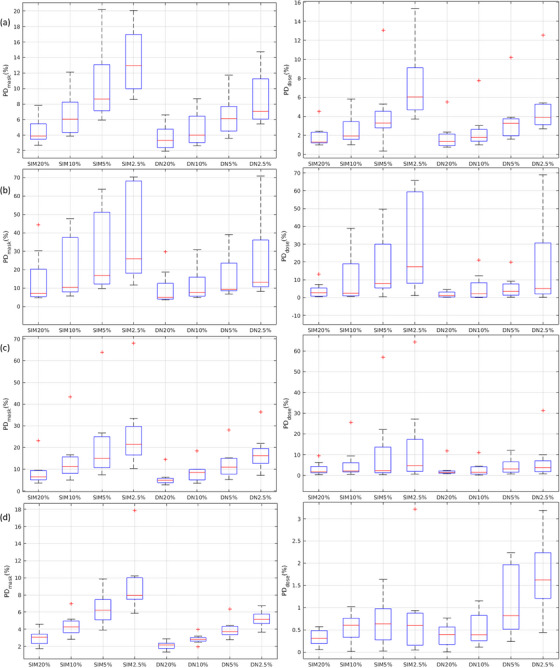
The box and whisker plots showing PD_mask_ (left) and PD_dose_ (right) for (a) spleen, (b) right kidney, (c) left kidney, (d) liver. The red line in each box represents the median of the distribution, whereas the top and bottom of each box represent the 25th and 75th percentile of the distribution, respectively. The whiskers extend to the minimum and maximum values for a data set. An outlier appears as a red plus sign.

Figure 2 demonstrates a NDCT image with volume CT dose index (CTDI_vol_) of 4.56 mGy, a LDCT image with CTDI_vol_ of 0.93 mGy, SPECT/NDCT fusion, simulated CT images at reduced dose levels (i.e., SIM_20%_, SIM_10%_, SIM_5%_, SIM_2.5%_), the denoising results of CT images at reduced dose levels (i.e., DN_20%_, DN_10%_, DN_5%_, DN_5%_), and the difference between CT images at reduced dose levels before and after denoising (i.e., diff_20%_, diff_10%_, diff_5%_, diff_2.5%_). The RMSE and PSNR for simulated CT images at reduced dose levels before and after denoising are shown in Table 1. Table 2 summarizes the mean and SD of HU values within segmented regions for spleen, right kidney, left kidney, and liver in NDCT, SIM_20%_, SIM_10%_, SIM_5%_, SIM_2.5%_, DN_20%_, DN_10%_, DN_5%_, DN_5%_. The degraded RMSE and PSNR values caused by dose reduction were effectively mitigated through denoising. Similar results were also observed in the SD values. Figure 3 demonstrates the NDCT, segmentation mask of NDCT (segNDCT), SPECT/NDCT fusion, dose rate maps, CT images at reduced dose levels before and after denoising, and their segmentation masks (before denoising: segSIM_20%_, segSIM_10%_, segSIM_5%_, segSIM_2.5%_; after denoising: segDN_20%_, segDN_10%_, segDN_5%_, segDN_2.5%_). The box and whisker plots in Figure 4 show PD_mask_ and PD_dose_ for spleen, right kidney, left kidney, and liver in simulated CT images at reduced dose levels before and after denoising. The mean and SD of PD_mask_ for each critical organ individually as well as collectively in SIM_20%_, SIM_10%_, SIM_5%_, SIM_2.5%_, DN_20%_, DN_10%_, DN_5%_, DN_5%_ are summarized in Table 3, while the corresponding results for PD_dose_ are summarized in Table 4. It was observed that greater dose reduction leads to increased CT segmentation errors, which, in turn, result in larger errors in dose rate measurements. Among the critical organs investigated, the right kidney exhibited the largest error, followed by the left kidney, spleen, and liver. Image denoising could ease the CT segmentation error, hence improving the accuracy in dose rate measurements.

**TABLE 1:** The RMSE and PSNR for simulated CT images at reduced dose levels before and after denoising.

	SIM_20%_	DN_20%_	SIM_10%_	DN_10%_	SIM_5%_	DN_5%_	SIM_2.5%_	DN_2.5%_
RMSE (HU)	25.70	17.07	38.05	21.06	55.37	25.98	79.89	32.63
PSNR (dB)	38.02	41.58	34.62	39.75	31.36	37.93	28.17	35.95

Abbreviations: PSNR, peak signal‐to‐noise ratio; RMSE, root mean square error.

**TABLE 2:** The mean and SD of HU values within segmented regions for the spleen, right kidney, left kidney, and liver.

	Spleen		Right kidney		Left kidney		Liver	
	Mean (HU)	SD (HU)	Mean (HU)	SD (HU)	Mean (HU)	SD (HU)	Mean (HU)	SD (HU)
NDCT	45.71	27.45	20.46	28.71	19.66	28.90	54.49	28.49
SIM_20%_	45.84	39.01	19.55	41.61	18.62	41.88	54.17	41.37
SIM_10%_	45.80	50.20	19.83	53.94	18.07	53.99	54.02	52.45
SIM_5%_	45.84	67.69	20.00	72.07	18.05	72.40	53.96	69.80
SIM_2.5%_	46.44	94.69	22.06	100.22	17.98	101.31	53.77	96.82
DN_20%_	45.57	24.05	19.19	26.62	18.55	26.80	54.49	25.29
DN_10%_	45.00	22.79	18.68	26.39	17.55	25.88	54.28	22.92
DN_5%_	45.29	21.94	18.02	26.21	17.01	25.66	54.77	22.18
DN_2.5%_	44.84	21.60	18.18	30.21	15.42	26.24	54.19	21.25

Abbreviations: HU, Hounsfield unit; SD, standard deviation.

**TABLE 3:** The mean and SD of PD_mask_ (%) for the spleen, right kidney, left kidney, liver, and all critical organs.

	Spleen		Right kidney		Left kidney		Liver		Total	
	Mean	SD	Mean	SD	Mean	SD	Mean	SD	Mean	SD
SIM_20%_	4.51	1.73	14.29	14.82	8.64	6.23	2.99	0.88	7.61	8.89
SIM_10%_	6.66	2.79	20.56	17.21	14.84	12.09	4.43	1.28	11.62	12.04
SIM_5%_	10.48	5.06	29.19	22.15	21.60	18.25	6.41	1.88	16.92	16.62
SIM_2.5%_	13.55	4.35	38.26	25.93	26.74	18.14	9.32	3.72	21.97	19.17
DN_20%_	3.68	1.70	9.50	9.61	5.83	3.71	2.09	0.48	5.28	5.71
DN_10%_	4.77	2.24	11.71	9.77	8.66	4.72	2.84	0.58	7.00	6.32
DN_5%_	6.49	2.67	16.03	12.95	12.61	7.19	3.99	1.09	9.78	8.66
DN_2.5%_	8.61	3.57	24.85	23.99	17.56	8.83	5.19	0.96	14.05	14.55

Abbreviation: SD, standard deviation.

**TABLE 4:** The mean and SD of PD_dose_ (%) for the spleen, right kidney, left kidney, liver, and all critical organs.

	Spleen		Right kidney		Left kidney		Liver		Total	
	Mean	SD	Mean	SD	Mean	SD	Mean	SD	Mean	SD
SIM_20%_	1.89	1.18	3.97	4.33	3.04	3.13	0.33	0.18	2.31	2.94
SIM_10%_	2.59	1.59	10.56	15.93	5.73	8.45	0.56	0.33	4.86	9.42
SIM_5%_	4.32	3.78	17.09	19.40	11.48	19.73	0.68	0.52	8.39	14.76
SIM_2.5%_	7.34	3.86	29.55	27.34	14.09	22.05	0.82	1.02	12.95	19.99
DN_20%_	1.88	1.57	1.74	1.63	2.74	3.75	0.38	0.26	1.69	2.25
DN_10%_	2.54	2.20	5.32	7.51	2.99	3.64	0.53	0.37	2.84	4.46
DN_5%_	3.71	2.76	5.64	6.47	4.41	4.05	1.14	0.80	3.72	4.22
DN_2.5%_	4.98	3.22	18.11	26.74	7.11	10.16	1.72	0.85	7.98	15.05

Abbreviation: SD, standard deviation.

## 4 DISCUSSION

The dosimetry workflow in ^177^Lu‐DOTATATE therapy consists of five general steps: (1) quantitative imaging such as SPECT/CT is performed at various time intervals following the administration of the radiopharmaceutical; (2) tumors and critical organs are delineated on SPECT/CT images to establish volumes of interest for dosimetry analysis; (3) the measured voxel values in the reconstructed SPECT images are converted into ^177^Lu activity; (4) the activities are integrated over time to obtain time‐integrated activity values; (5) the time‐integrated activity values in tumor and critical organs are converted into absorbed doses. Hence, dose estimates can be influenced by variability in segmentation, decay correction, curve fitting, and integration methods.^35^, ^36^, ^37^ Segmentation of the relevant anatomic structures is an important step in ^177^Lu DOTATATE therapy because it affects both activity and mass estimates. Manual segmentation is a tedious and time‐consuming task, which is also susceptible to inter‐ and intra‐observer variability. According to Uribe et al., the median total time to complete the dosimetry workflow for ^177^Lu DOTATATE therapy was 89 min, with segmentation being the most time‐consuming step, requiring a median of 43 min.^35^ Auto‐segmentation is likely to reduce contouring inconsistencies while also saving time. In recent years, several semantic segmentation models were proposed.^38^, ^39^ Among them, nun‐Net has been externally validated and won several open‐sourced medical image segmentation challenges in the 2018 Medical Decathlon Segmentation Challenge.^40^, ^41^ Since nnU‐Net automatically configures the segmentation framework itself, including preprocessing, network architecture, training, and postprocessing for any new task, the characteristics of the dataset is crucial to model performance.^42^ The TotalSegmentator model pipeline was built on the nnU‐Net architecture and trained on a dataset containing 1204 CT examinations that were acquired using various imaging protocols and scanners from multiple manufacturers.^23^ Tsanda et al. have investigated the segmentation performance of TotalSegmentator on CT images at reduced dose levels.^43^ According to their results, the Dice score decreased by no more than 3% at a 20% dose level, inspiring us to explore the potential of reducing CT dose involved in ^177^Lu DOTATATE dosimetry workflow by utilizing TotalSegmentator.

Based on our results, the mean ± SD of PD_mask_ in SIM_20%_ was 7.61 ± 8.89% for all critical organs collectively. With regard to PD_dose_, the corresponding results were 2.31 ± 2.94%. However, as dose reduction increased, CT image segmentation gradually deteriorated, which in turn reduced the dosimetry accuracy of ^177^Lu DOTATATE therapy. The mean ± SD of PD_mask_ for all critical organs collectively was 11.62 ± 12.04% in SIM_10%_, 16.92 ± 16.62% in SIM_5%_, and 21.97 ± 19.17% in SIM_2.5%_. With regard to PD_dose_, the corresponding results were 4.86 ± 9.42%, 8.39 ± 14.76%, and 12.95 ± 19.99%. Convolutional neural networks (CNN) are emerging as powerful tools for medical image denoising.^24^, ^25^ DenseNet employs inter‐block connections to reuse convolution features and uses skip connections to enhance feature transfer without the problem of gradient vanishing, so it was chosen for image denoising in this work.^29^, ^44^, ^45^ Based on visual inspection, the overall texture difference between NDCT and DN_20%_ was minimal, but it became more noticeable as the dose level decreased. As seen in Table 2, the maximum difference in mean HU value between NDCT and DN_20%_ was 1.27 HU (right kidney), which was 1.78 HU for DN_10%_ (right kidney), 2.65 HU for DN_5%_ (left kidney), 4.24 HU for DN_2.5%_ (left kidney). On the other hand, the SD values measured from CT images at reduced dose levels were substantially decreased after denoising. Reducing statistical fluctuations could improve CT image segmentation, thereby enhancing the dosimetry accuracy of ^177^Lu DOTATATE therapy. The mean ± SD of PD_mask_ for all critical organs collectively were 5.28 ± 5.71% in DN_20%_, 7.00 ± 6.32% in DN_10%_, 9.78 ± 8.66% in DN_5%_, and 14.05 ± 14.55% in DN_2.5%_. With regard to PD_dose_, the corresponding results were 1.69 ± 2.25%, 2.84 ± 4.46%, 3.72 ± 4.22%, 7.98 ± 15.05%. In peptide receptor radionuclide therapy, the kidneys are typically the dose‐limiting organ, with a threshold of 23 Gy.^46^, ^47^ However, due to bio‐distribution of ^177^Lu DOTATATE, spleen often receives higher doses than other critical organs, followed by the right kidney, left kidney, and liver.^34^, ^48^ The dosimetry accuracy in DN_20%_ decreased by no more than 3% for each critical organ individually as well as collectively, and the overall texture difference between NDCT and DN_20%_ was barely noticeable. These findings suggested that the investigated deep learning methods have the potential to reduce CT dose down to 20% of normal dose while maintaining dosimetry accuracy for patients undergoing serial SPECT/CT imaging during ^177^Lu DOTATATE therapy.

## 5 CONCLUSION

As deep learning methods become more integrated into clinical practice, understanding both their capabilities and limitations is vital. Based on our results, the dosimetry accuracy of critical organs in ^177^Lu DOTATATE therapy decreased by no more than 4% at a 20% dose level when using TotalSegmentator for organ segmentation. However, an increase in dose reduction degraded CT image segmentation gradually, which in turn deteriorated the dosimetry accuracy of ^177^Lu DOTATATE therapy. Improving CT image quality through denoising could enhance ^177^Lu DOTATATE dosimetry, making it a valuable tool to support CT dose reduction for patients undergoing serial SPECT/CT imaging during treatment.

## AUTHOR CONTRIBUTIONS

*Conceptualization*: Hung‐Te Yang, Kuan‐Yin Ko, and Ching‐Ching Yang. *Methodology*: Hung‐Te Yang, Kuan‐Yin Ko, and Ching‐Ching Yang. *Software*: Hung‐Te Yang, Ching‐Ching Yang. *Formal analysis*: Ching‐Ching Yang. *Writing‐review and editing*: Hung‐Te Yang, Kuan‐Yin Ko, and Ching‐Ching Yang. *Visualization*: Hung‐Te Yang, Kuan‐Yin Ko; *Supervision*: Ching‐Ching Yang.

## CONFLICT OF INTEREST STATEMENT

The authors have no competing interests to declare that are relevant to the content of this article.
